# Characterization of bacterial and viral pathogens in the respiratory tract of children with HIV-associated chronic lung disease: a case–control study

**DOI:** 10.1186/s12879-024-09540-5

**Published:** 2024-06-26

**Authors:** Prince K. Mushunje, Felix S. Dube, Courtney Olwagen, Shabir Madhi, Jon Ø Odland, Rashida A. Ferrand, Mark P. Nicol, Regina E. Abotsi, The BREATHE study team, Tsitsi Bandason, Ethel Dauya, Tafadzwa Madanhire, Elizabeth L. Corbett, Katharina Kranzer, Edith D. Majonga, Victoria Simms, Andrea M. Rehman, Helen A.Weiss, Hilda Mujuru, Dan Bowen, Louis-Marie Yindom, Sarah L. Rowland-Jones, Trond Flaegstad, Tore J. Gutteberg, Jorunn Pauline Cavanagh, Trym Thune Flygel, Evegeniya Sovarashaeva, Jessica Chikwana, Gugulethu Newton Mapurisa, Carmen Gonzalez-Martinez, Robina Semphere, Brewster Wisdom Moyo, Lucky Gift Ngwira, Slindile Mbhele

**Affiliations:** 1 Department of Molecular and Cell Biology & Institute of Infectious Diseases and Molecular Medicine, University of Cape Town, Cape Town, South Africa; 2 School of Medicine, University of Lusaka, Lusaka, Zambia; 3 South Africa Medical Research Council Vaccines and Infectious Diseases Analytics Research Unit, Faculty of Health Sciences, University of the Witwatersrand, Johannesburg, South Africa; 4 Infectious Diseases and Oncology Research Institute, Faculty of Health Sciences, University of the Witwatersrand, Johannesburg, South Africa; 5 Faculty of Biosciences and Aquaculture, Nord University, Bodø, Norway; 6 International Research Laboratory for Reproductive Ecotoxicology (IL RET), The National Research University Higher School of Economics, Moscow, Russia; 7 School of Health Systems and Public Health, Faculty of Health Sciences, University of Pretoria, Pretoria, South Africa; 8 Biomedical Research and Training Institute, Harare, Zimbabwe; 9 Clinical Research Department, London School of Hygiene and Tropical Medicine, London, UK; 10 Marshall Centre, Division of Infection and Immunity, School of Biomedical Sciences, Faculty of Health and Medical Sciences, University of Western Australia, Perth, Australia; 11 Department of Pharmaceutical Microbiology, School of Pharmacy, University of Health and Allied Sciences, Ho, Ghana

**Keywords:** *S. pneumoniae*, *M. catarrhalis*, *H. influenzae*, Pneumococcal serotypes, Human rhinovirus, Obliterative bronchiolitis, Africa

## Abstract

**Introduction:**

Chronic lung disease is a major cause of morbidity in African children with HIV infection; however, the microbial determinants of HIV-associated chronic lung disease (HCLD) remain poorly understood. We conducted a case–control study to investigate the prevalence and densities of respiratory microbes among pneumococcal conjugate vaccine (PCV)-naive children with (HCLD +) and without HCLD (HCLD-) established on antiretroviral treatment (ART).

**Methods:**

Nasopharyngeal swabs collected from HCLD + (defined as forced-expiratory-volume/second < -1.0 without reversibility postbronchodilation) and age-, site-, and duration-of-ART-matched HCLD- participants aged between 6–19 years enrolled in Zimbabwe and Malawi (BREATHE trial-NCT02426112) were tested for 94 pneumococcal serotypes together with twelve bacteria, including *Streptococcus pneumoniae* (SP), *Staphylococcus aureus* (SA), *Haemophilus influenzae* (HI), *Moraxella catarrhalis* (MC), and eight viruses, including human rhinovirus (HRV), respiratory syncytial virus A or B, and human metapneumovirus, using nanofluidic qPCR (Standard BioTools formerly known as Fluidigm). Fisher's exact test and logistic regression analysis were used for between-group comparisons and risk factors associated with common respiratory microbes, respectively.

**Results:**

A total of 345 participants (287 HCLD + , 58 HCLD-; median age, 15.5 years [IQR = 12.8–18], females, 52%) were included in the final analysis. The prevalence of SP (40%[116/287] *vs.* 21%[12/58], *p* = 0.005) and HRV (7%[21/287] *vs.* 0%[0/58], *p* = 0.032) were higher in HCLD + participants compared to HCLD- participants. Of the participants positive for SP (116 HCLD + & 12 HCLD-), 66% [85/128] had non-PCV-13 serotypes detected. Overall, PCV-13 serotypes (4, 19A, 19F: 16% [7/43] each) and NVT 13 and 21 (9% [8/85] each) predominated. The densities of HI (2 × 10^4^ genomic equivalents [GE/ml] *vs.* 3 × 10^2^ GE/ml, *p* = 0.006) and MC (1 × 10^4^ GE/ml *vs.* 1 × 10^3^ GE/ml*, p* = 0.031) were higher in HCLD + compared to HCLD-. Bacterial codetection (≥ any 2 bacteria) was higher in the HCLD + group (36% [114/287] *vs.* (19% [11/58]), (*p* = 0.014), with SP and HI codetection (HCLD + : 30% [86/287] *vs.* HCLD-: 12% [7/58], *p* = 0.005) predominating. Viruses (predominantly HRV) were detected only in HCLD + participants. Lastly, participants with a history of previous tuberculosis treatment were more likely to carry SP (adjusted odds ratio (aOR): 1.9 [1.1 -3.2], *p* = 0.021) or HI (aOR: 2.0 [1.2 – 3.3], *p* = 0.011), while those who used ART for ≥ 2 years were less likely to carry HI (aOR: 0.3 [0.1 – 0.8], *p* = 0.005) and MC (aOR: 0.4 [0.1 – 0.9], *p* = 0.039).

**Conclusion:**

Children with HCLD + were more likely to be colonized by SP and HRV and had higher HI and MC bacterial loads in their nasopharynx. The role of SP, HI, and HRV in the pathogenesis of CLD, including how they influence the risk of acute exacerbations, should be studied further.

**Trial registration:**

The BREATHE trial (ClinicalTrials.gov Identifier: NCT02426112, registered date: 24 April 2015).

**Supplementary Information:**

The online version contains supplementary material available at 10.1186/s12879-024-09540-5.

## Introduction

In 2019, over 2.8 million children and adolescents were living with HIV globally, 90% in sub-Saharan Africa [1]. Respiratory infections remain the most common manifestation of HIV among these children and adolescents [2, 3]. The scale-up of antiretroviral therapy (ART) has increased survival so that growing numbers of children are entering adulthood. In addition, ART has resulted in a reduction in the rate of respiratory disorders, including tuberculosis and lymphocytic interstitial pneumonitis [4–7]. However, studies in sub-Saharan Africa revealed that approximately 30% of HIV-infected older children experience chronic respiratory symptoms, including chronic cough and reduced tolerance to exercise, which often leads to presumptive tuberculosis treatment [8]. The clinical and radiological picture of this chronic lung disease is consistent with small airway disease, predominantly constrictive obliterative bronchiolitis [9].

The pathogenesis of this condition is incompletely understood. It is speculated that HIV-induced chronic inflammation and dysregulated immune activation may play a role [10–12]. A previous study of older children with HIV-associated chronic lung disease (HCLD) conducted by our group demonstrated that there was increased inflammatory activation in children with HCLD (HCLD +) compared to their HIV-infected counterparts without HCLD (HCLD-) [13]. In the same cohort, there was an association between the carriage of specific bacteria in the nasopharynx and HCLD [14]. Specifically, we observed that older children with HCLD were more likely to be colonized with *Streptococcus pneumoniae* (SP) and *Moraxella catarrhalis* (MC) than their HCLD- counterparts [14]. The study utilized bacterial culture, which is limited by viability and a narrow spectrum of culturable bacterial species. Although we observed that SP was associated with HCLD, we did not investigate the specific serotypes that may be involved in this condition, which is important to inform pneumococcal immunization. Furthermore, the prevalence of respiratory viruses was also not studied.

Viruses facilitate bacterial infections in the host through various mechanisms, including damaging the respiratory epithelium, modifying the immune response, and altering cell membranes [15]. Coinfection of viruses and bacteria leads to increased bacterial load, thus making individuals more susceptible to complications related to upper respiratory tract infections [16]. Prior to COVID-19, respiratory syncytial virus, influenza virus and human rhinovirus (HRV) were the most common causative agents of upper respiratory infection and have been linked to exacerbations of COPD [17, 18], asthma development [17], and severe bronchiolitis in children [19–21].

To overcome these limitations, we investigated the prevalence of respiratory pathogens in both HCLD + and HCLD- participants using real-time quantitative polymerase chain reaction (qPCR) to detect and quantify a large number of bacterial and viral targets and elucidate common SP serotypes (94 serotypes). We also assessed clinical and sociodemographic factors associated with microbial carriage and density.

## Materials and methods

### Study design, population, and setting

This case–control study was nested within the BREATHE trial (ClinicalTrials.gov Identifier: NCT02426112, registered date: 24 April 2015) investigating whether azithromycin therapy could improve lung function and reduce the risk of exacerbations among children with HCLD [22]. BREATHE was a two-site, double-blinded, placebo-controlled, individually randomized trial conducted in Harare (Zimbabwe) and Blantyre (Malawi). The study setting, population, and trial procedures are described elsewhere [22–24]. Briefly, we enrolled perinatally HIV-infected participants aged 6 – 19 years with HCLD. HCLD was defined as a forced expiratory volume in 1 s (FEV1) *z* score < -1, with no reversibility (< 12% improvement in FEV1 after salbutamol 200 µg inhaled using a spacer) [22]. A group of perinatally HIV-infected children without HCLD (FEV1* z* score > 0) was also recruited at the same time as the enrollment of trial participants using frequency matching for site, sex, age, and duration of ART to serve as a comparison group for pathogenesis studies. Both groups were on ART for at least six months. All participants were most likely not vaccinated due to the introduction of PCV13 in 2012 in Zimbabwe [25] and in Malawi in 2011 [26], making them ineligible for vaccination at that time because of their older age. Swabs were collected between June 1, 2016 and September 31, 2019. Sociodemographic data and clinical history were recorded through an interviewer-administered questionnaire.

### Nasopharyngeal swab collection

Nasopharyngeal swabs were collected at baseline from all participants using sterile flocked flexible nylon swabs (Copan Italia, Brescia, Italy). Swabs were immediately immersed in 1 mL PrimeStore® Molecular Transport Medium (MTM) (Longhorn Vaccines & Diagnostics LLC, Bethesda, USA), transported on ice and stored at -80 °C at the diagnostic laboratory at each site. PrimeStore® MTM was used because it is a medium optimized for transporting and storing samples for molecular analyses; it also inactivates potential pathogens and stabilizes nucleic acids [27]. The samples were batched and transported on dry ice to Cape Town, South Africa, where they were stored at -80 °C until further processing.

### Total nucleic acid extraction

Total nucleic acid (TNA) extraction for microbial identification was conducted on NP swabs stored in Primestore® MTM. Briefly, the samples were thawed and vortexed for 10 s, and 400 µl aliquots were transferred into ZR BashingBead™ Lysis Tubes containing 0.5 mm beads (catalog no. ZR S6002–50, Zymo Research Corp., Irvine, CA, United States) for the mechanical lysis steps. Lysis was conducted on a Qiagen Tissue lyser LTTM (Qiagen, FRITSCH GmbH, Idar-Oberstein, Germany) for 5 min at 50 Hz, followed by centrifugation (Eppendorf F-45–30-11, Merck KgaA, Darmstadt, Germany) for 1 min at 10,000 rpm (10,640 g). The supernatants (250 µl) were extracted using the QIAsymphony® DSP Virus/Pathogen Kit (Qiagen GmbH, Hilden, Germany) on the QIAsymphony SP/AS instrument (Qiagen GmbH, Hilden, Germany) following the manufacturer’s instructions. The total nucleic acid was eluted in 70 µl DNA elution buffer into the Elution Microtube (Qiagen GmbH, Hilden, Germany) and immediately stored at -80 °C until further analysis.

### Real-time qPCR using the biomark HD system (Fluidigm assay)

Nanofluidic qPCR testing was performed at the WITS-VIDA Research Unit, Witwatersrand University, Johannesburg, South Africa as previously described [28, 29]. Briefly, all extracts were tested for 94 SP serotypes together with 12 bacterial species (SP, HI, MC, *Staphylococcus aureus* [SA], *Neisseria lactamica*, *Neisseria meningitidis*, *Streptococcus pyogenes*, *Bordetella pertussis*, *Bordetella holmesii*, *Klebsiella pneumoniae*, *Acinetobacter baumanii* and *Streptococcus oralis*), 6 HI serotypes and 8 viruses (respiratory syncytial virus A and B, human rhinovirus, influenza A and B, human parainfluenza 1 and 3, and human metapneumovirus). Furthermore, all samples were previously cultured for SP, HI, MC and SA as described elsewhere [14]. These microbial targets (Table S1) included on the nanofluidic panel might be associated with HCLD and are the most frequent pathobionts in the nasopharynx. A detailed list of these microbial targets can be found in the supplementary material (Table S1). For SP, positive samples were defined as those with a Cycle of quantification (Cq) value ≤ 36 for each serotype-specific qPCR target and positive for both *Lyt*A and *Pia*B. Negative samples were defined as those with Cq values ≥ 36 for each target.

The bacterial or serotype densities were determined following the method outlined by Downs et al*.* [28]. Briefly, culture controls and synthetic double-stranded DNA (dsDNA) template gene fragments (gBlocks) were included in the assay as external calibrators, reported as copy numbers or gene equivalents, respectively. A DNA library was prepared for the targeted pneumococcal serotypes or other bacterial species at an average concentration ranging from 10^3^ to 10^4^ CFU/ml. For assay-sets meeting the defined efficiency criteria (90–110%), the relative quantification of bacterial density was determined by extrapolating using the linear equation derived from standard curves of the calibrators (control strains and gBlocks with known densities), employing the equation and reported as log 10 genomic equivalents/ml:

$${Density=10}^{\frac{Cq-c}mx\frac{extraction\;volume}{elution\;volume}}$$

### Data management and statistical analysis

Clinical and sociodemographic data were electronically captured using Google NexusTM tablets (Google, Mountain View, CA, USA) running OpenDataKit software, managed on Microsoft Access databases (Microsoft, Redmond, WA, USA) and analyzed using Stata ((StataCorp, College Station, TX). Comparisons between groups were performed with the Student T test or Mann–Whitney U test for continuous data and chi-squared or Fisher’s exact tests for categorical data where appropriate with no further adjustment of multiplexity. Multivariate logistic regression, adjusting for age category, duration of ART, site, sex, height-for-age, HIV viral suppression, history of TB treatment, Medical Research Council dyspnea score and ART regimen, was used to investigate the factors associated with microbial carriage and density. The following were excluded from the multivariate model because of colinearity: Enrollment BMI-for-age z score, weight-for-age z score, and CD4 count. A *p* value of less than 0.05 was considered statistically significant.

## Results

### Clinical and sociodemographic characteristics

The study included 345 participants, HCLD + (*n* = 287) and HCLD- (*n* = 58), with a median age [IQR] of 15.5 (12.8 – 18.0) years and 52% (180/345)] female (Table 1). The median BMI-for-age-*z* score for the HCLD + group was lower compared to the HCLD- group (-1.1 *vs* -0.4), *p* < 0.001. A higher proportion of the participants from the HCLD + group were previously treated for tuberculosis (31% *vs.* 12%, *p* = 0.001), stunted (49% *vs*. 29%, *p* = 0.009) and underweight (52% *vs*. 14%, *p* < 0.001) compared to the HCLD- group. A higher number of HCLD + participants were on a second-line ART (protease inhibitor-based) regimen (25% *vs.* 10%, *p* = 0.01) compared to HCLD-. Ten percent of HCLD + participants compared to HLCD- (2%) participants had an MRC dyspnea score of 3 or above. None of the participants reported smoking.

**Table 1:** Baseline characteristics of participants in the HCLD + and HCLD- groups

Characteristics		HCLD + [% (n/N)]	HCLD- [% (n/N)]	*P*^b^
**Sociodemographic**				
Site	Zimbabwe, % (n/N)	72% (208/287)	76% (44/58)	0.746
	Malawi	28% (79/287)	24% (14/58)	
Sex	Male	51% (147/287)	31% (18/58)	**0.006**
	Female	49% (140/287)	69% (40/58)	
Age (years)	Median (IQR)	15.4 (12.9 – 19.9)	15.7 (12.4 – 18.1)	0.861
Age groups	6 – 12 years	26% (74/287)	29% (17/58)	0.373
	13 – 16 years	43% (123/287)	31% (18/58)	
	17 – 19 years	31% (90/287)	40% (23/58)	
Currently attending school		81% (231/284)	86% (50/58)	0.454
Same age as most children in class ^a^		34% (79/233)	26% (13/50)	0.442
Repeated ≥ 1 school grade,		58% (162/281)	40% (23/58)	**0.014**
**Anthropometric:**				
BMI for age-z score	Median (IQR)	-1.1 (-1.8, -0.2)	-0.14 (-0.8, 0.7)	** < 0.001**
Height for age-z score	< − 2 (Stunted)	49% (140/287)	29% (17/58)	**0.009**
Weight for age-z score	< − 2 (Underweight)	52% (148/287)	14% (8/58)	** < 0.001**
**Current Drugs:**				
Taking cotrimoxazole prophylaxis ^a^		91% (259/285)	87% (48/55)	0.454
Antiretroviral Regimen	NNRT-base-1st line	75% (214/287)	90% (52/58)	**0.010**
	PI-base 2nd line	25% (73/287)	10% (6/58)	
**HIV Clinical parameters**				
Age at HIV diagnosis (years),	median (IQR)	7.9 (4.4 – 10.5)	7.1 (4.1 – 9.8)	0.377
Age at ART initiation (years),	median (IQR)	8.5 (5.9 – 11.7)	8.4 (5.0 – 11.1)	0.398
Duration on ART (years),	median (IQR)	6.4 (3.9 – 8.6)	7.3 (4.7 – 9.0)	0.219
Duration on ART^a^	> 6 months to < 2 years	9% (26/280)	3% (2/58)	0.341
	2 to < 4 years	17% (48/280)	21% (12/58)	
	4 to < 6 years	21% (58/280)	16% (9/58)	
	6 years or more	53% (148/280)	60% (35/58)	
CD4 count categories (Cells/mm^3^)	< 200	12% (33/287)	10% (6/58)	0.687
	200–500	29% (84/287)	24% (14/58)	
	> 500	59% (170/287)	66% (38/58)	
Viral load (VL) suppression^a^	VL < 1000 copies/mL	54% (153/285)	66% (38/58)	0.191
**Respiratory status**				
Hospitalization for chest problems in the last 48 weeks		2% (6/287)	0% (0/58)	0.595
Previously treated for TB^a^		31% (88/286)	12% (7/58)	**0.001**
Has asthma^a^		3% (9/286)	0% (0/58)	0.366
FEV1* z* score	Median (IQR)	-1.9 (-2.46, -1.47)	0.61 (0.26 – 0.83)	** < 0.001**
MRC dyspnea score^a^	1	54% (156/287)	81% (46/57)	**0.006**
	2	36% (103/287)	17% (10/57)	
	3	6% (18/287)	2% (1/58)	
	4	3% (8/287)	0% (0/57)	
	5	1% (2/287)	0% (0/57)	

*Abbreviations: HCLD* HIV-associated chronic lung disease, *TB* Tuberculosis, *FEV1* Forced expiratory volume in 1 s, *IQR* Interquartile range, *MRC* Medical Research Council

^a^Participants with missing responses were excluded from that variable; *p*^*b*^*,* Fisher’s exact test

### Prevalence and densities of selected nasopharyngeal microbes in participants with and without HCLD

The prevalence and median densities of selected microbes detected in the nasopharynx of HCLD + and HCLD- participants are summarized in Table 2. The prevalence of SP colonization was significantly higher in the HCLD + group (40%, 116/278) compared to the HCLD- group (21%, 12/58; *p* = 0.005). However, there were no statistically significant differences between the two groups in colonization prevalence of pneumococcal serotypes covered by the 13-valent pneumococcal conjugate vaccine (PCV13 vaccine types, VT) or those not covered (non-vaccine types, NVT).

**Table 2:** The prevalence and density of microbes present in the nasopharyngeal swabs of HIV-infected children with and without HCLD

Microorganism	Microbial prevalence			Microbial density (GE/ml)		
	HCLD + group (*n* = 287)	HCLD- group (*n* = 58)	*p*^*a*^	HCLD + group (Median)	HCLD- group (Median)	*p*^*b*^
**Bacterial species**						
* Streptococcus pneumoniae* (SP)	40% (116)	21% (12)	**0.005**	6 × 10^4^	1 × 10^4^	0.259
* Staphylococcus aureus* (SA)	6% (17)	5% (3)	0.999	3 × 10^2^	2 × 10^2^	0.773
* Moraxella catarrhalis* (MC)	26% (75)	16% (9)	0.095	1 × 10^4^	1 × 10^3^	**0.031**
* Haemophilus influenzae* (HI)	43% (124)	33% (19)	0.147	2 × 10^4^	3 × 10^2^	**0.006**
* Haemophilus influenzae* type B	0% (0)	2% (1)	0.999	-	*1 × 10^3^	-
* Streptococcus oralis*	3% (8)	2% (1)	0.999	8 × 10^2^	*2 × 10^3^	0.699
* Neisseria lactamica*	2% (6)	0% (0)	0.595	2 × 10^3^	-	-
* Streptococcus pyogenes*	2% (5)	0% (0)	0.594	3 × 10^6^	-	-
**Virus**						
Respiratory syncytial virus A	0.4% (1)	0% (0)	0.999	* 3 × 10^2^	-	-
Respiratory syncytial virus B	0.4% (1)	0% (0)	0.999	* 62	-	-
Human rhinovirus	7% (21)	0% (0)	**0.032**	1 × 10^4^	-	-

*CLD* Chronic lung disease, *GE* Genomic Equivalents

^a^Fisher's exact test; did not adjust for multiple testing

^*b*^ Mann‒Whitney U test

^*^Actual microbial density obtained from one participant

**Fig. 1:**
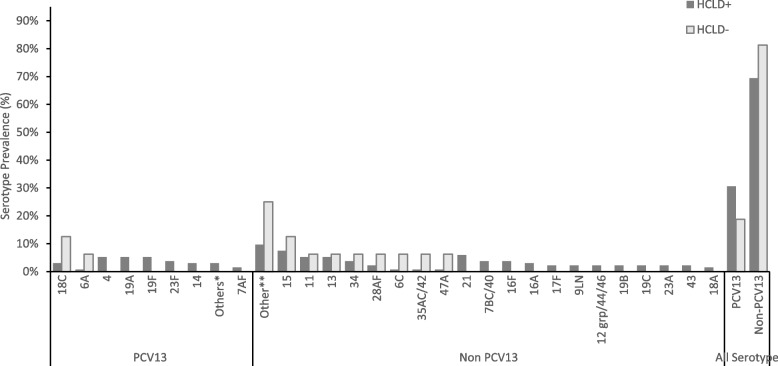
Pneumococcal serotypes recovered from nasopharyngeal swabs of HCLD + and HCLD- participants. Abbreviations: PCV, polysaccharide-conjugated vaccine; n, number of swabs serotyped using the fluidigm assay from the HCLD + group (*n* = 116) and HCLD- group (*n* = 12). Denominator for prevalence is the total number of SP serotypes grouped into PCV 13 and non-PCV 13 serotypes. Others* HCLD + group: PCV13 serotype [3 (0.7%), 5 (0.7%), 6B (0.7%), 9AV (0.7%), 4 (5.2%), Others**: HCLD + group non-PCV 13 serotype [18B (0.7%), 19 atypical (0.7%), 20 (0.7%), 23B (1.5%), 27 (0.7%), 25AF/38 (0.7%), 45 (0.7%), 29 (0.7%), 31 (0.7%), 33C (1.5%), 38 (0.7%)]; HCLD- group non-PCV13 serotype [10A (6.3%), 10B (6.3%), 22A (6.3%), 33B (6.3%)]. 15: HCLD + group non-PCV 13 serotype [15AF (3.7%), 15BC (2.2%), 15like (1.5%)]; HCLD- group non-PCV13 serotype [15like (6.3%)]. 11: HCLD + group non-PCV 13 serotype [11AD [3.7%), 11E (1.5%)]; HCLD- group non-PCV13 serotype [11E (6.3%)]

Of the 128 participants colonized with SP (116 HCLD + , 12 HCLD-), 66% (85/128) carried NVT serotypes, while 34% (43/128) carried PCV13 VT serotypes (Fig. 1). A total of 150 pneumococcal serotypes was detected in the 128 participants colonized with SP including 134 serotypes from the HCLD + group and 16 from the HCLD- group (Fig. 1). Multiple serotypes were detected in 14% (16/116) of HCLD + participants and 17% (2/12) of the HCLD- participants colonized with SP.

NVT serotypes predominated over PCV13 VTs in both groups, accounting for 69% (93/134) in HCLD + and 81% (13/16) in HCLD- (Fig. 1). While not statistically significant (*p* = 0.398), the prevalence of PCV13 VT serotypes trended higher in HCLD + (31%, 41/134) compared to HCLD- (19%, 3/16).

The most common PCV13 VT serotypes in both groups were 4 (16%, 7/44), 19F (16%, 7/44), 19A (16%, 7/44), and 18C (14%, 6/44). The predominant NVTs were 13 and 21 (8% each, 8/106). There were no statistically significant differences in serotype-specific colonization prevalence between HCLD + and HCLD- groups. Likewise, the median densities of the composite NVT and PCV13 VT serotypes did not differ significantly between the two groups (Figure S1). The overall median serotype density across all samples was 8.8 genomic equivalents/ml.

There were no significant differences in the colonization prevalence of any other bacteria tested. Despite there being no difference in the colonization prevalence for both HI and MC, the mean log density was higher in the HCLD + ( 2 × 10^4−^gene equivalents [GE]/ml & 1 × 10^4^ GE/ml) compared to the HCLD- (3 × 10^2^ GE/ml; *p* = 0.006 & 0.5 × 10^3^ GE/ml; *p* = 0.031,) groups, respectively (Table 2). There was no significant difference in the mean log densities between the groups for the other tested bacteria. There was a low prevalence of the viruses detected amongst the group, with HRV (7% [21/287] *vs*. 0% [0/58], *p* = 0.032) detected in the HCLD + group only. The bacterial species *Klebsiella pneumoniae*, *Neisseria meningitidis*, *Actinobacter baumanii*, *Bordetella pertussis/holmesii*, and viruses influenza A, influenza B, human parainfluenza type 1 & 3, and human metapneumovirus were not detected in any participants.

### Nasopharyngeal bacterial and viral co-colonization in participants with and without HCLD

Bacterial and viral co-colonization detected in HIV-infected participants with or without HCLD is summarized in Table 3 and Table S2. Bacterial detection (any) was significantly higher in HCLD + (61% [175/287]) than in HCLD- (43.1% [25/58]) (*p* = 0.013). Moreover, the concurrent carriage of multiple bacterial species was higher in the HCLD + group (35.9% [103/287]) than in the HCLD- group (19% [11/58]) (*p* = 0.014). The most frequent bacteria detected concurrently with SP included SP were HI (HCLD + : 30% [87/287]) *vs.* HCLD-: 12.1% [7/58], *p* = 0.013) and MC (HCLD + : 23.3% [67/287] *vs.* HCLD-: 12.1% [7/58],* p* = 0.078). Viruses were detected only in the HCLD + group (8% [23/287]), with viral and bacterial co-colonization reported in 6.6% (19/287) of HCLD + participants. To determine whether the concurrent detection of bacteria in HCLD + participants was due to a true interaction or simply by chance, we compared the observed and expected values based on marginal probabilities. The results showed that the co-colonization of SP with HI (Observed [86/287] vs Expected [50.1/287], *p* < 0.001); SP with MC (Observed [60/287] vs Expected [30.3/287]], *p* < 0.001) and MC with HI (Observed [54/287] vs Expected [32.4/287], *p* < 0.001]) was a result of true interactions with HCLD (Table S3).

**Table 3:** Bacterial and viral co-colonization in study participants at baseline

	HCLD + group % (n/N)	HCLD—group % (n/N)	***P***^***a***^
**Bacterial detection**			
Single bacterial species	61% (175/287)	43.1% (25/58)	**0.013**
At least 2 bacterial species	35.9% (103/287)	19% (11/58)	**0.014**
> 2 bacterial species	21.3% (61/287)	12.1% (7/58)	0.147
**Bacterial co-colonization**			
SP & SA	2.4% (7/287)	3.4% (2/58)	0.651
SP & HI	30% (86/287)	12.1% (7/58)	**0.005**
SP & MC	20.9% (60/287)	12.1% (7/58)	0.146
* SA* & HI	2.1% (6/287)	1.7% (1/58)	1.000
* SA* & MC	1.1% (3/287)	3.5% (2/58)	0.198
MC & HI	18.8% (54/287)	12.1% (7/58)	0.261
SP & other bacterial species ^a^	4.9% (14/287)	3.5% (2/58)	1.000
MC & other bacterial species ^a^	3.1% (9/287)	1.7% (1/58)	1.000
HI & other bacterial species ^a^	5.2% (15/287)	3.5% (2/58)	0.748
SA & other bacterial species	3.1% (9/287)	0% (0/58)	0.366
**Virus detection**			
Single virus	8.0% (23/287)	0% (0/58)	**0.019**
≥ 2 viruses	-	-	-
**Viral and bacterial co-detection**			
Single virus + single bacterial species	6.6% (19/287)	0% (0/58)	0.053
Single virus + multiple bacterial species	5.6% (16/287)	0% (0/58)	0.084

Other bacteria ^a^; bacteria analyzed as one group (*Klebsiella pneumoniae*, *Neisseria meningitidis*, *Acinetobacter baumanii* and *Bordetella pertussis/holmesii*, *HI* type B, *S. oralis*, *N. lactamia* & *S. pyogens*); *p*^*a*^, Fisher's exact test

### Factors associated with carriage of selected bacteria at baseline in participants with HCLD

The results of the univariate and multivariate analyses of the clinical and sociodemographic factors associated with the carriage of SP and SA are displayed in Table 4, while those for HI and MC are shown in Table 5. On multivariate analysis, participants previously treated for TB (adjusted odds ratio were more likely to carry SP (aOR): 1.9 [1.1 -3.2], *p* = 0.021) or HI (aOR: 2.0 [1.2 – 3.3], *p* = 0.011). Participants on ART for ≥ 2 years (aOR: 0.3 [0.1 – 0.8], *p* = 0.005) and living in Zimbabwe (aOR: 0.5 [0.3 – 0.9], p = 0.026) were less likely to carry HI (Table 5). Similarly, MC carriage was less likely in participants who had been on ART for ≥ 2 years (aOR: 0.4 [0.1 – 0.9], *p* = 0.039) (Table 5). Participants who were attending school were more likely to carry MC (aOR: 2.5 [1.0 -6.4], *p* = 0.050) (Table 5).

**Table 4:** Univariate and multivariate analysis of factors associated with nasopharyngeal SP and SA colonization at baseline

Variable	Total observation (*N* = 345)	SP colonization					SA colonization				
	% (n)	Colonized % (n)	OR [95% CI]	***P***	Adjusted OR [95% Cl)	***p***^**a**^	Colonized, % (n)	OR [95% CI]	***p***	Adjusted OR [95% Cl)	***p***^**a**^
**Group**											
HCLD-	17% (58)	21% (12)	Reference	**0.006**	Reference	**0.028**	5% (3)	Reference	0.824	Reference	0.896
HCLD +	83% (287)	40% (116)	2.6[1.3–5.1]		2.2 [1.1–4.8]		6% (17)	1.2[0.3–4.1]		0.9[0.2–4.6]	
**Sex**											
Male	48% (165)	35% (58)	Reference	0.473	Reference	0.158	7% (12)	Reference	0.266	Reference	0.114
Female	52% (180)	39% (70)	1.2[0.8–1.8]		1.4[0.9–2.3]		4% (8)	0.6[0.2–1.5]		0.4[0.1–1.2]	
**Study site**											
Malawi	27% (93)	45% (42)	Reference	0.061	Reference	0.183	8% (7)	Reference	0.406	Reference	0.293
Zimbabwe	73% (252)	34% (86)	0.6[0.4 -1.0]		0.7[0.4–1.2]		5% (13)	0.7[0.3—1.7]		2.0[0.5–7.7]	
**Baseline age category (years)**											
6 – 12	26% (91)	44% (40)	Reference	**0.048**	Reference	0.325	7% (6)	Reference	0.167	Reference	0.983
13 – 16	41% (141)	40% (56)	0.8[0.5–1.4]		0.8[0.4–1.4]		8% (11)	1.2 [0.4–3.4]		1.8[0.5–6.6]	
17 – 19	33% (113)	28% (32)	0.5[0.3–0.9]		0.5[0.2–1.0]		3% (3)	0.4[0.1–1.6]		0.5 [0.1 -2.4]	
**Currently attending school**											
No	18% (63)	33% (21)	Reference	0.482			2% (1)	Reference	0.163		
Yes	82% (281)	38% (107)	1.2[0.3–1.2]				6% (18)	4.2[0.6–32]			
**History of Hospitalization for chest problems**											
No	98% (339)	37% (124)	Reference	0.154	Reference	0.124	6% (19)	Reference	0.278	Reference	-
Yes	2% (6)	67% (4)	3.5[0.4 – 19]		5.9[0.6–58]		17% (1)	3.4[0.4 – 30]		Omitted	
**Ever treated for TB**											
No	72% (249)	33%(81)	Reference	**0.006**	Reference	**0.021**	6% (16)	Reference	0.431	Reference	0.312
Yes	28% (95)	48% (46)	2.0 [1.2 -3.2]		1.9[1.1–3.2]		4% (4)	0.6[0.2–2.0]		0.5[0.1–1.8]	
**Ever treated for asthma**											
No	97% (277)	40% (111)	Reference	0.359			6% (16)	Reference	0.514		
Yes	3% (9)	56% (5)	1.9[0.5–7.1]				11% (1)	2.0[0.2–17]			
**Duration on ART (years)**											
> 6 months to < 2 years	8% (28)	54% (15)	Reference	0.064	Reference	0.188	18% (1)	Reference	0.693	Reference	0.542
2 to < 4 years	18% (60)	48% (29)	0.7[0.3- 1.8]		1.4[0.5–4.3]		5% (3)	1.4[0.1- 14]		1.7[0.1–35]	
4 to < 6 years	20% (67)	37% (25)	0.6[0.3—1.5]		0.5[0.2–1.4]		9% (6)	2.7[0.3- 23]		5.7[0.3–103]	
6 years or more	54% (183)	31% (56)	0.4[0.2—0.8]		0.5[0.2–1.4]		27% (10)	1.6[0.3- 12]		3.2[0.2–53]	
**CD4 count (Cells/ml)**											
< 200	11% (39)	41% (16)	Reference	0.591			8% (3)	Reference	0.850		
200–500	28% (98)	38% (37)	0.9[0.4–1.9]				5% (5)	0.6[0.1–2.8]			
> 500	60% (208)	36% (75)	0.8[0.4–1.6]				6% (12)	0.7[0.2- 2.7]			
**Baseline viral load**											
Unsuppressed	45% (154)	37% (57)	Reference	0.871	Reference	0.962	8% (13)	Reference	0.096	Reference	0.066
Suppressed	55% (191)	37% (70)	0.9[0.6–1.5]		1.0[0.6–1.6]		4% (7)	0.4[0.2- 1.2]		2.7[0.9–7.8]	
**ART regimen**											
NNRT-base-1st line	77% (266)	36% (95)	Reference	0.328	Reference	0.794	6% (15)	Reference	0.818	Reference	0.472
PI-base 2nd line	23% (79)	42% (33)	0.8 [0.5–1.3]		1.2[0.6–1.9]		6% (5)	1.1[0.4 -3.2]		152[0.3- 5.0]	
**Cotrimoxazole prophylaxis**											
No	10% (33)	39% (13)	Reference	0.77	Reference	0.333	6% (2)	Reference	0.963	Reference	0.592
Yes	90% (307)	37% (113)	0.9[0.4–1.9]		0.7[0.3–1.5]		6% (18)	1.0[0.2–4.4]		1.8[0.2–14.9]	
**Baseline weight-for-age-z score**											
Not underweight	55% (189)	33% (63)	Reference	0.552			5% (10)	Reference	0.658		
Underweight	45% (156)	42% (65)	1.4[0.9–2.2]				6% (10)	1.2[0.5–3.0]			
**Baseline height-for-age-z score**											
Not stunted	54% (188)	34% (63)	Reference	0.131	Reference	0.366	6% (12)	Reference	0.611	Reference	0.337
Stunted	46% (157)	41% (65)	1.4[0.9–2.2]		1.3[0.8–2.0]		5% (8)	0.8[0.3–2.0]		0.6[0.2–1.7]	

*Abbreviations: NNR* Nonnucleoside reverse transcriptase inhibitor, *No.* Number of participants, *IQR* Interquartile range, *TB* Tuberculosis, *PI* Protease inhibitor, *FEV1* Forced expiratory volume in one second*, %* Row percentages are presented in the cells

Participants with missing responses were excluded from that variable: *currently attending school, n* = *1; antiretroviral regimen, n* = *1; history of TB, n* = *1*; current cough, *n* = 1; cotrimoxazole prophylaxis, *n* = 5; viral load, *n* = 2; and duration on ART, *n* = 7

^a^Variables included in the multivariate logistic regression are participant group, age category, duration of ART, site, sex, height-for-age-z score, viral suppression, history of TB treatment, MRC dyspnea score and ART regimen

**Table 5:** Univariate and multivariate analysis of factors associated with nasopharyngeal HI and MC colonization at baseline

Variable	Total observations (*N* = 345)	HI colonization					MC colonization (*n* = 84)				
	% (n)	Colonized % (*n* = 143)	OR [95% CI]	***p***	Adjusted OR [95% Cl)	***p***^**a**^	Colonized % (*n* = 84)	OR [95% CI]	***P***	Adjusted OR [95% Cl)	***p***^**a**^
**Group**											
HCLD-	17% (58)	33% (19)	Reference	0.143	Reference	0.326	16% (9)	Reference	0.074	Reference	0.273
HCLD +	83% (287)	43% (124)	1.6 [0.9–2.8]		1.4 [0.7- 2.7]		26% (75)	1.9[0.9—4.1]		1.6[0.7–3.5]	
**Sex**											
Male	48% (165)	41% (67)	Reference	0.761	Reference	0.457	23% (38)	Reference	0.585	Reference	0.523
Female	52% (180)	42% (76)	1.1 [0.5–1.6]		1.2 [0.7–1.9]		25% (46)	1.1[0.7—1.9]		1.2[0.7–2.0]	
**Study site**											
Malawi	27% (93)	54% (50)	Reference	**0.005**	Reference	**0.026**	30% (28)	Reference	0.136	Reference	0.152
Zimbabwe	73% (252)	37% (93)	0.5[0.3–0.8]		0.5 [0.3 – 0.9]		22% (56)	0.7[0.4—1.1]		0.6[0.4–1.2]	
**Baseline age category (years)**											
6 – 12	26% (91)	45% (41)	Reference	0.275	Reference	0.425	12% (11)	Reference	0.326	Reference	0.839
13 – 16	41% (141)	44% (62)	1.0[0.6- 1.6]		1.0[0.5–1.7]		27% (38)	0.7[0.3–1.3]		1.2[0.6–2.4]	
17 – 19	33% (113)	35% (40)	0.7[0.4–1.2]		0.8[0.3- 1.5]		19% (22)	1.0[0.6–1.9]		1.4[0.6–3.4]	
**Currently attending school**											
No	18% (63)	37% (23)	Reference	0.368			13% (8)	Reference	**0.011**	Reference	**0.050**
Yes	82% (281)	43% (120)	1.3[0.7–2.3]				27% (76)	2.5[1.2–5.6]		2.5[1.0- 6.4]	
**History of Hospitalization for chest problems**											
No	98% (339)	41% (140)	Reference	0.670	Reference	0.502	24% (81)	Reference	0.171	Reference	0.133
Yes	2% (6)	5% (3)	1.4[0.3–7.1]		1.9 [0.5 -12.7]		5% (3)	3.2[0.616]		4.2[0.6–27]	
**Ever treated for TB**											
No	72% (249)	38% (94)	Reference	**0.029**	Reference	**0.011**	22% (56)	Reference		Reference	0.328
Yes	28% (95)	51% (48)	1.7[1.1–2.7]		2.0 [1.2–3.3]		29% (28)	1.4[0.8–2.4]	0.185	1.4[0.7–2.7]	
**Ever treated for Asthma**											
No	97% (277)	43% (118)	Reference	0.167			61% (72)	Reference			
Yes	3% (9)	67% (6)	2.7[0.7–11]				33% (3)	1.4[0.3–5.8]	0.624		
**Duration on ART (years)**											
> 6 months to < 2 years	8% (28)	71% (20)	Reference	**0.005**	Reference	**0.005**	43% (12)	Reference	**0.021**	Reference	
2 to < 4 years	18% (60)	43% (26)	0.3[0. -0.8]		0.3[0.1–1.1]		22% (13)	0.4[0.1–1.0]		0.5[0.1–1.6]	
4 to < 6 years	20% (67)	42% (28)	0.3[0.1–0.7]		0.2[0.1–0.8]		19% (13)	0.3[0.1–0.8]		0.3[0.1–1.0]	**0.039**
6 years or more	54% (183)	36% (65)	0.2[0.1–0.5]		0.3[0.1–0.9]		24% (44)	0.4[0.2–1.0]		0.6[0.2–1.7]	
**CD4 count (Cells/ml)**											
< 200	11% (39)	59% (23)	Reference	0.065			33% (13)	Reference	0.293		
200–500	28% (98)	39% (38)	0.4[0.2–0.9]				20% (20)	0.5[0.2–1.2]			
> 500	60% (208)	39% (82)	0.5[0.8–2.7]				25% (51)	0.6[0.3–1.4]			
**Baseline viral load**											
Unsuppressed	45% (152)	42% (64)	Reference	0.813	Reference	0.803	23% (35)	Reference		Reference	0.662
Suppressed	55% (191)	41% (78)	0.9 [0.6–1.5]		1.1 [0.7–1.7]		25% (48)	3.3[0.2–54.8]	0.664	0.8[0.5–1.5]	
**ART regimen**											
NNRT-base-1st line	77% (266)	42% (111)	Reference	0.846	Reference	0.628	23% (62)	Reference		Reference	0.466
PI-base 2nd line	23% (79)	41% (32)	1.0[0.6–1.6]		0.9[0.5–1.5]		28% (22)	1.3[0.7–2.2]	0.414	1.2[0.7–2.3]	
**Cotrimoxazole prophylaxis**											
No	10% (33)	42% (14)	Reference	0.878	Reference	0.413	24% (8)	Reference		Reference	0.644
Yes	90% (307)	41% (126)	0.9[0.5 -2.0]		0.7 [0.3 -1.5]		24% (75)	1.1[0.4–2.3]	0.981	0.8[0.5–1.3]	
**Baseline weight-for-age-z score**											
Not underweight	55% (189)	42% (80)	Reference	0.715			23% (43)	Reference			
Underweight	45% (156)	40% (63)	0.9[0.6–1.4]				26% (41)	1.2[0.7–2.0]	0.446		
**Baseline height-for-age-z score**											
Not stunted	54% (188)	39% (73)	Reference	0.280	Reference	0.658	25% (47)	Reference		Reference	0.105
Stunted	46% (157)	45% (70)	1.3[0.8–1.9]		1.1 [0.7—1.8]		24% (37)	0.9[0.6–1.5]	0.757	4.8[0.5–1.3]	

*Abbreviations: NNRT* Nonnucleoside reverse transcriptase inhibitor, *No.* Number of participants, *IQR* Interquartile range, *TB* Tuberculosis, *PI* Protease inhibitor, *FEV1* Forced expiratory volume in one second*, %* Row percentages are presented in the cells

Participants with missing responses were excluded from that variable: *currently attending school, n* = *1; antiretroviral regimen, n* = *1; history of TB, n* = *1*; current cough, *n* = 1; cotrimoxazole prophylaxis, *n* = 5; viral load, *n* = 2; and duration on ART, *n* = 7

^a^Variables included in the multivariate logistic regression are participant group, age category, duration of ART, site, sex, height-for-age-z score, viral suppression, history of TB treatment, MRC dyspnea score and ART re

## Discussion

In this study, we used quantitative PCR to determine the prevalence and density of bacterial and viral carriage in HIV-infected African children. As previously shown [14], microbial colonization was more frequently detected in HCLD + than HCLD- participants, with the former more likely to carry SP or HRV. Strikingly, viruses (predominantly HRV) were detected only in HCLD + children. Moreover, we observed that HCLD + participants had a higher HI and MC density than their HCLD- counterparts. The prevalence and densities of all SP serotypes tested were similar between the two groups, with more of the recovered SP serotypes (79%) being non-PCV 13. Study participants with a history of previous tuberculosis treatment were more likely to carry SP or HI, while those who used ART for ≥ 2 years were less likely to carry HI and MC. Furthermore, those living in Zimbabwe were less likely to carry HI.

The prevalence of HI in the current study in both HCLD + (43%) and HCLD- (33%) participants was higher than that observed in our previous study of the same cohort by Abotsi et al. [14] (12% and 5%, respectively). Similar studies conducted in India [30] and Zambia [31] in HIV-infected children observed similar prevalence (26% and 29%, respectively) to our current study (33%). The discrepancy in results could be attributed to the more sensitive PCR detection method used in our study compared to the culture method employed in previous studies as well as age and pathological differences.

Furthermore, HCLD + participants showed a higher density of HI than their counterparts. Previous studies have associated HI carriage in participants with other lung diseases, including asthma [32], bronchiectasis [33, 34] and chronic obstructive pulmonary disease [35–37]. HI has been identified as a biomarker for predicting the response to azithromycin treatment in adults with persistent uncontrolled asthma [32]. It has also been associated with negative outcomes in children suffering from respiratory viral infections [38], including hospitalization among RSV-positive children [39]. The bacterium’s ability to invade host epithelial cells, evade host defense mechanisms, form biofilms and survive as an intracellular pathogen contributes to its pathogenic nature [40], which may suggest an important role that it may play in HCLD + pathogenesis. However, further studies are needed to further elucidate this observation.

The prevalence of carriage of SA in this study (HCLD + [6%] and HCLD- [5%]) was markedly lower than that observed using bacterial culture in the same cohort (HCLD + [23%] and HCLD- [19%]) [14]. This difference in prevalence may be related to the efficiency of the nucleic acid extraction method used. The extraction of nucleic acids requires extended and vigorous lysis steps for some bacterial species (gram-positive such as SA) compared to others (gram-negative such as HI and MC) [41]; however, for this study, although we used a rigorous extraction protocol incorporating bead-beating, the inherent complexity and resilience of the SA bacterial cell wall may have contributed to the low yield. This underscores the need for tailored approaches and ongoing refinement of extraction methods to include enzymatic lysis alternatives such as lysostaphin or lysozyme in NP swabs DNA extraction for enhanced yield. Additionally, carriage may have been influenced by the efficiency of the annealing of PCR primers [42].

High SP, HI and MC density in the nasopharynx has been associated with respiratory infections in children [43, 44]. This is consistent with our study, where a higher HI and MC density was observed in HCLD + participants than in their HCLD- counterparts. The HCLD + participants may have a chronic lung infection, as evidenced by the isolation of bacteria from their sputum in our previous study [14]. Microbiota dominated by *Haemophilus*, *Moraxella* or *Neisseria* species are associated with chronic lung diseases, including chronic obstructive pulmonary disease and asthma [45–48]. Bhadriraju et al*.* [40] observed that HIV-infected children with a sputum bacteriome dominated by *Haemophilus*, *Moraxella* or *Neisseria* species were 1.5 times more likely to have HCLD than those with *Streptococcus* or *Prevotella* spp. [40]. These bacterial genera were also associated with enhanced inflammatory effects [40]. Interestingly, we detected *Neisseria* species (*N. lactamica*) in HCLD + participants only. Taken together, these findings support the important role of HI and MC in HCLD.

Our observation of a higher SP carriage in the HCLD + group than in the HCLD- group is consistent with our culture-based study of the same cohort [14]. Furthermore, SP carriage in the HCLD- participants (21%) is comparable to studies of HIV-infected children in South Africa (22.2%) [49] and Cambodia (17.6%) [42, 50]. Nevertheless, the prevalence is higher than that observed in children living with HIV in Ethiopia (10.3%) [51] and lower than that in studies from Ghana (27.1%) and Tanzania (81%) [52]. The differences in SP prevalence observed between studies can again be related to differences in the age of participants—younger children have a higher carriage prevalence[31, 52], socioeconomic factors [53] and the geographical location of the participants.

The prevalence of PCV 13 serotypes and densities in both study groups (HCLD + : 30.4% and HCLD-: 16.7%) did not differ significantly. The most prevalent PCV-13 serotypes were serotypes 4 (15.9%), 19F (15.9%), 19A (15.9%) and 18C (14%). A study of HIV-infected children in Malawi [54] reports 19F and 6A among the most predominant serotypes. The relatively high prevalence of serotype 19A in PCV-vaccinated children has been suggested by Kamng’ona et al*.* [54] to occur due to an inversion in the *rmlD* gene at the CPS locus. This may downregulate the *rmlD* gene on the CPS locus, causing an altered 19A capsule [55] that is not recognized by the PCV vaccine.

There was a higher prevalence of non-PCV13 serotypes in both the HCLD + (69% [93/134]) and HCLD- (81% [13/16]) compared to PCV13 serotypes. Similar findings were reported in Malawi [54], Nigeria [56] and Ghana [57]. We assume that community herd protection from vaccinated siblings, neighbors, and playmates may be responsible for the low prevalence of vaccine-type serotypes in our cohort. Continued surveillance of SP and its non-PCV 13 serotypes is warranted to inform future vaccine formulation and roll-out strategies, especially in this vulnerable population.

There is evidence suggesting a relationship between SP and other pathogens co-colonizing the nasal and pharyngeal mucosae [58]. Our analysis, based on expected values, revealed a positive positive association between SP with HI and MC carriage in participants with CLD, which is consistent with previous reports by Madhi et al*.* [59] in HIV-infected South African children. A similar positive association between SP and HI was observed in a study of HIV-infected children in India (median age was 6.5 years, IQR [4.5 – 9]) [30]. HI modulates the expression of SP genes in biofilms primarily by upregulating the type IV pilus structural protein, which is essential for adhesion and stability [60, 61]. Polymicrobial infections involving these microbes and others have been demonstrated to exacerbate higher disease severity and increased tolerance to antimicrobials [62, 63]. Further studies are warranted to comprehensively understand the mechanisms underlying these interactions and their implications for CLD + pathogenesis and treatment strategies.

The major risk factors associated with the development of pneumococcal disease are demographic (age and sex) and immune status (CD4 count and HIV viral load) [64]. We observed no association between these common factors and most bacterial species, including SP. This is supported by previous studies that reported a lack of association between CD4 count and the prevalence of pneumococcal carriage [31, 65, 66]. A longer period on an ART regimen (two years or more) was associated with reduced carriage of MC and HI. Similar results were obtained from a study among HIV-infected adults in Brazil [67]. ART therapy could help reduce the risk of infection and carriage through immune reconstitution [67].

The presence of viruses increases bacterial adherence, and the difference in the prevalence of viruses in HCLD + *vs* HCLD- children may partially explain the increased HI and MC densities we observed. Our findings are consistent with a study by Binks et al*.* [44], who reported an increased SP and HI density during coinfection with respiratory viruses within the nasopharynx of Australian children with otitis media. However, no significant difference in the bacterial load was detected in SP from the HCLD + and HCLD- groups. Viruses expose the host to bacterial infection through various mechanisms, including the destruction of the respiratory epithelium, modulation of innate defenses and alteration of cell membranes, which facilitates bacterial adherence [15]. Ishizuka et al*.* [68], in their in vitro studies, observed increased SP adherence to epithelial cells after infection with HRV. They suggested that this observation may explain why pneumonia develops following an HRV infection [68]. Interestingly, we found no association between any virus (HRV, RSVA and RSVB) and the prevalence or density of carriage of SP or other bacterial species tested. This contrasts with several in vitro and in vivo studies that have suggested that respiratory virus infection increases bacterial adherence and subsequent bacterial superinfection within the nasopharynx [68–70]. This discrepancy may be explained by the few viruses we detected due to the limited sample size, especially in the HCLD- group.

HRV is responsible for most upper respiratory tract infections and their complications, including bronchitis [15]. In a study of HIV-infected children in India [71], HRV was the most prevalent virus in these participants when asymptomatic. The GABRIEL multicenter case‒control study in Africa and Asia also found HRV in healthy control groups of pneumonia childhood studies [72]. In contrast, our study detected HRV (7%) in only HCLD + participants. Notably, RSV infection was uncommon, consistent with previous studies conducted in Africa and Asia (PERCH case‒control studies [73] and DCHS case‒control studies [74]), which showed its infrequency except during acute respiratory infections.

In conclusion, our study findings indicate that HCLD + participants were more commonly colonized by any of the bacteria tested compared to HCLD- participants. Specifically, the HCLD + group had a higher prevalence of carriage of SP bacteria, as well as a higher density of MC and HI bacteria. Interestingly, viruses, particularly HRV, were detected only in the HCLD + group. Moreover, our research revealed that previous treatment for tuberculosis was positively associated with the carriage of HI or SP bacteria among study participants. On the other hand, being a female participant was found to be less likely to be associated with SA carriage. Additionally, longer periods on the ART regimen were associated with reduced carriage of HI or MC bacteria. Our study sheds light on the quantitative information on microbial carriage and nasopharyngeal carriage of viruses and serotypes of HI and SP in children with HCLD + . The limitations of our study included a small sample size of HCLD participants, potentially impacting the statistical power and generalizability of our findings. Additionally, our statistical analysis did not correct for multiplexity. While one approach to address multiplexity is adjusting the p-value threshold to α = 0.05 divided by the number of tests conducted, this method may result in a considerable reduction in the number of statistically significant findings. Therefore, there is a need for more comprehensive studies in this population to further investigate the role of SP, HI, MC, and HRV in the pathogenesis of CLD and the underlying mechanisms behind these bacterial associations.

### Supplementary Information

Supplementary Material 1.

## Data Availability

The datasets used and analyzed during the current study are available from Felix Dube (sizwe.dube@uct.ac.za) on reasonable request and ethical approval.

## Abbreviations

ART: Antiretroviral therapy
AZM: Azithromycin
HCLD: HIV-associated chronic lung disease
HCLD +: HIV-infected participants with HIV-associated chronic lung disease
HCLD-: HIV-infected participants without HIV-associated chronic lung disease
NP: Nasopharyngeal
HI: *Haemophilus influenza*
MC: *Moraxella catarrhalis*
SA: *Staphylococcus aureus*
SP: *Streptococcus pneumoniae*
HRV: Human rhinovirus
RSV: Respiratory syncytial virus
NVT: Non PCV 13 vaccine serotypes
VT: PCV 13 vaccine serotypes
