# Supplementary material for: Characterization of bacterial and viral pathogens in the respiratory tract of children with HIV-associated chronic lung disease: a case–control study
Source: BMC Infect Dis. 2024 Jun 26;24:637. doi: 10.1186/s12879-024-09540-5 (PMC11201860; doi:10.1186/s12879-024-09540-5)
Supplement: Supplementary file 1 — Supplementary Material 1. [file 12879_2024_9540_MOESM1_ESM.docx]

## Supplementary information

Table S1. Oligonucleotide primers and probe sequences for the ‘Fluidigm’ qPCR reaction-set (96 assay-sets) for detection of 94 pneumococcal serotypes, 15 other bacterial and one fungal species

| **Assay-set name** | **Forward primer (5' to 3')** | **Reverse primer (5' to 3')** | **Probe (5' to 3')** | **Gene Target** | **Reference** |
| --- | --- | --- | --- | --- | --- |
| S. pneumoniae Serotype 1 † | CGTGCGGTAATTGAAGCTATGA | TGTGGCCCCAGCAACTCT | FAM-TGCTTGCCCTTGTATAGGGT-NFQ | wch*D* | Azzari *et al.* [1] |
| S. pneumoniae Serotype 2 ‡ | TTATGGACTGGCTGATGGTTCTC | AAATCCTGACCCAATAATAGCCTTT | FAM-AGGTCAACG/ZEN/TATTGGAAC  TCTTAGAAATTGGGAAA-IABkFQ | wzy | Pholwat *et al.* [2] |
| S. pneumoniae Serotype 3 † | GGTCAGCAGAAAGTATGCATTGG | TCGTTTATCCAGGGTCTGATGA | VIC-TATTGGATGTGGT  TTATCGTGAAGA-NFQ | tnp | Azzari *et al.* [1] |
| S. pneumoniae Serotype 4 † | TGGGATGACATTTCTACGCACTA | CCGTCGCTGATGCTTTATCA | FAM-TCCTATTGGATG  GTTAGTTGGTGA-NFQ | wzy | Azzari *et al.* [1] |
| S. pneumoniae Serotype 5 † | TTACGGGAGTATCTTATGTCTTTAATGG | CAGCATTCCAGTAGCCTAAAACTAGA | VIC-TTGTCTCAGCAACT  CTATTTGGCTGTGGG-NFQ | wzy | Azzari *et al.* [1] |
| S. pneumoniae Serogroup 6A/B/C/D  (F/G/H) † | AAGTTTGCACTAGAGTATGGGAAGGT | ACATTATGTCCRTGTCTTCGATACAAG | VIC-GTTCTGCCCTGAGCAACTGG-NFQ | wci*P* | Azzari *et al.* [1] |
| S. pneumoniae Serotype 6A/C ‡ | CATTGCTAGAGATGGTTCCTTC  AGTTGATATTGATAAAGATTCG  GGAGACATGTCCAAACTGGC | CGATACAAGACCAGTTGC | FAM-GTTTGCACT/ZEN/AGAGTA  TGGGAAGGTGTTGT-IABkFQ | wci*P*α | Downs *et al*. [3] |
| S. pneumoniae Serotype 6C/D † | TTGGGATGATTGGTCGTATTAG | CTCTTCAATTAGTTCTTCAGTTCG | FAM-CCACGCAATTCGCCATC-NFQ | wci*N*_β_ | Azzari *et al.* [1] |
| S. pneumoniae Serogroup 7A/F † | GATGGCATGTGGCAAACCA | TTTGCCCTCCTTAATCATTTCAC | FAM-TTGGCTATCGGCATGGTGGT-NFQ | wcw*H* | Azzari *et al.* [1] |
| S. pneumoniae Serogroup 7B/C/40 ‡ | TCCAGATATAGTCATTCCCAATCAG | AAAGAAGGTAAATCCCATGATGAATT | FAM-TCCCTCATTATCGATTA  CTGACCCACCA-BHQ1 | wcx*U* | Pholwat *et al.* [2] |
| S. pneumoniae Serotype 8 † | CCACTCATCAGTTTCCCATATGTTT | TCAATAATTGAAGAAGCGAACGTT | FAM-TGATGGCAGAT  GGGTTGGGACGAG-NFQ | wzx | Azzari *et al.* [1] |
| S. pneumoniae Serogroup 9A/L/N/V ‡ | TGGAATGGGCAAAGGGTAGTA | TCGGTTCCCCAAGATTTTCTC | FAM-TTAATCATGCTAACGG  CTCATCGA-BHQ1 | mna*A* | Olwagen *et al.* [4] |
| S. pneumoniae Serogroup 9A/V‡ | AGGTATCCTATATACTGCTTTAGG | CGAATCTGCCAATATCTGAAAG | FAM-ACACATTGA  CAACCGCTACA-BHQ1 | wzx | Pholwat *et al.* [2] |
| S. pneumoniae Serogroup 9L/N ‡ | CGTGGAATTTTCTATACTGCAATAGG | CTACTGCTACGATACCATATTCTACAG | FAM-CAATTCTTAG  CCGGATTCTCTC-BHQ1 | wzx | Pholwat *et al.* [2] |
| S. pneumoniae Serogroup 10A/B † | CCTCTCCTATCAACTAT  TACTCATTATACTACCT | AATAACCATAAGTCC  CTAGATCATTCAAAG | VIC-TCATTACAACTCCCTA  TGTGACACGGGTCTTTT-NFQ | wcr*D* | Azzari *et al.* [1] |
| S. pneumoniae Serotype 10B † | AAATATGAGATTGGT  AAGGAATATTCTGG | GTCTTTTCACTTAAACGAATTCCATTC | FAM-AACGGATTCCAATGC  ACTCGGTAACT-NFQ | wcr*D* | Pholwat *et al.* [2] |
| S. pneumoniae Serogroup 10C/F † | CGAGTTATGGATGTTCTTATTGGC | CCCAACCCCACTCTGTATTG | FAM-ACAGGGCAAGACTGT  GAATATTGTTCCA-NFQ | wcj*G* | Sakai *et al.* [5] |
| S. pneumoniae Serogroup 11A/B/C/ D/F/(E) † | ACCGCATTTCTTATCGCACTATATT | TCTCCTTACCATCAAACATGTTAATCA | FAM-TGAATCAGTCTGACCGTTT-NFQ | wzx | Olwagen *et al.* [4] |
| S. pneumoniae Serogroup 11A/D † | CGGCCCAGCTACATTTATGG | TGATCATTCACATGCTCACCAA | FAM-AAATACCAATAGTTGT  TCCGAGATTAAAGAAGT-NFQ | wch*K* | Pholwat *et al.* [2] |
| S. pneumoniae Serogroup 11B/C † | TCAAATTTGGCGTATTGCTTATCA | TGATTATGAGCATAGTTGATCCCC | FAM-TCCGTGGCAAGATTCT  GGTGCTAAG-NFQ | wzy | Sakai *et al.* [5] |
| S. pneumoniae Serotype 11F † | TGGTCCAGCTACTTTTATGGC | TGATCATTCACATGCTCCCC | VIC-ACTCCAATAGTTGTTC  CGAGGCAAAAGA-NFQ | wch*K* | Pholwat *et al.* [2] |
| S. pneumoniae Serogroup 12A/B/F/44/  46 † | GATTATTCGCTTGCCTCTTCATG | ATAGCCGAAATAAGCTTTCCAGAA | FAM-ATTTGTAAGCG  GACGTGCGATT-NFQ | mna*B* | Azzari *et al.* [1] |
| S. pneumoniae Serotype 12B † | GGTTGCTGATCAAAAGGTCTATG | AGGTTCAAAGTAAGATTTTTAGCAA | FAM-AGATAAAAATCTTTCC  AAATCATCAAAGTGA-NFQ | wzx | Pholwat *et al.* [2] |
| S. pneumoniae Serotype 13 † | TCGGATTTAGTAGTAACCCCATTGA | TTCTTGATTGAGGATGCATTTCC | VIC-AGTAGTAAGAG  ATCATATTCAAG-NFQ | wzy | Olwagen *et al.* [4] |
| S. pneumoniae Serotype 14 † | CGACTGAAATGTCACTAGGAGAAGAT | AATACAGTCCATCAA  TTACTGCAATACTC | VIC-TGTCATTCGTTTGCCA  ATACTTGATGGTCTC-NFQ | wch*L* | Azzari *et al.* [1] |
| S. pneumoniae Serogroup 15A/B/C/F ‡ | TTGAATCAGGTAGATTGATTTCTGCTA | CTCTAGGAATCAAAT  ACTGAGTCCTAATGA | FAM-CTCCGGCTTT  TGTCTTCTCTGT-BHQ1 | wzx | Azzari *et al.* [1] |
| S. pneumoniae Serogroup 15A/F ‡ | CGTTATTTAGTGAATTGCTATACTC | TCCCTGCAGAATAAGAATCTAC | FAM-TACtGCtGCtGCcAACA-BHQ1 | wzy | Messaoudi *et al.*  [6] |
| S. pneumoniae Serogroup 15B/C ‡ | TTTGCTACAGGTTTTAGTATTGAG | AAAGCAATATAAGAGGTATAGTTGG | FAM-CGCtACaATcATcCGCT-BHQ1 | wzy | Messaoudi *et al.*  [6] |
| S. pneumoniae Serotype 16A † | GCTAGCAGGAACTTTTCTAGGG | TCCCTGTCCAAATCCGAAAC | FAM-CCCACGGGATGAATC  CATTATGGCG-NFQ | wcx*R* | Sakai *et al.* [5] |
| S. pneumoniae Serotype 16F † | GCAACTGGTATTTTTGATATTGGAGAA | CAAAGGAATGCCATGCCATA | FAM-AAAATGCTAAC  TTCGTTGGAGG-NFQ | wzy | Olwagen *et al.* [4] |
| S. pneumoniae Serotype 17A † | TGATTATGTCATTCGATTGCTTGG | AAATCCTAAAATTCCTGTTTGAAAAGC | FAM-ATTATGGGCGTG  GGTTACCGTAGG-NFQ | wzy | Sakai *et al.* [5] |
| S. pneumoniae Serotype 17F † | GTAAAGATTTCATGT  CCTATAAGGGAGAA | AGGCGTCCCTGTTTATGAGAAG | FAM-TTGTACATGGTCTGGATTT-NFQ | Wzx | Olwagen *et al.* [4] |
| S. pneumoniae Serogroup 18A/B/C/F ‡ | CCTGTTGTTATTCACGCCTTACG | TTGCACTTCTCGAATAGCCTTACTC | FAM-AACCGTTGGC  CCTTGTGGTGGA-BHQ1 | wzy | Azzari *et al.* [1] |
| S. pneumoniae Serogroup 18B/C/F ‡ | CAGGATTTCTAACTCTGATTGAA | AGCAAAATCTAACGTCCAGAG | FAM-CTTGTATGCTTATGG  TCTTTTCGATTA-BHQ1 | wci*X*_BCF_ | Downs *et al*. [3] |
| S. pneumoniae Serogroup 18C/F ‡ | CCAAATTGGAGTGTTTTACAA  AGTATTAGCTCGATTTGCTGT  ACACGTCGACGCTTCAATTTCAGG | TCTTTCAAATACAACT  CTTAGATTTCCTTGTG | FAM-TGagtTTATTGATAATttcC-BHQ1 | wci*X*_CF_ | Downs *et al*. [3] |
| S. pneumoniae Serotype 18F:  S. pneumoniae Serogroup 16F/18F/28AF ‡ | TGGTTTCGGACTCTTTCGTGG | CTAAGATAGAAACTCCTTGTCCAATG | FAM-GGTTGTACGTGGAAT  CGGATTTGGTC-BHQ1 | wcx*M* | Downs *et al*. [3] |
| S. pneumoniae Serotype 19A † | TTCGACGACGTATCAGCTTCA | TCATTGAGAGCCTTAACCTCTTCA | VIC-ACCCAAAACGGTTG  ACGCATTATACT-NFQ | wzy | Azzari *et al.* [1] |
| S. pneumoniae Serogroup 19B/F † | GGTCATGCGAGATACGACAGAA | TCCTCATCAGTCCCAACCAATT | VIC-ACCTGAAGGAGTAG  CTGCTGGAACGTTG-NFQ | wzy | Azzari *et al.* [1] |
| S. pneumoniae Serotype 19F † | CAGGTTCGGGAAATTGCAA | ATCTCTGCGCCATAAGCAATG | VIC-AGAAGTGGCAGATGATT-NFQ | wzh | Downs *et al*. [7] |
| S. pneumoniae Serotype 20 † | AAAGATACTGGCTGAGGAGCTATCTATT | AGTCAAAAGTACTCAA  CCATTCTGATATATTC | VIC-AGGATAAGGTCTACT  TTGTGGGAGTTC-NFQ | wci*L* | Azzari *et al.* [1] |
| S. pneumoniae Serotype 21 † | CCATTTGAAGGACCAGTTGTTG | AAAAAGCCACTATCAGGAATACCAA | FAM-AATGGCATTGCTTCGTAAA-NFQ | wzy | Olwagen *et al.* [4] |
| S. pneumoniae Serogroup 22A/F † | TCTATTAAATAACCC  ATTGGAATTGAAACG | TCGCAATTGAAGACCACATAAACTG | FAM-TCCGTAATTCGCTTA  TGGGCACATTCTCCA-NFQ | wcw*V* | Azzari *et al.* [1] |
| S. pneumoniae Serotype 22F ‡ | GAAGATTGTCCACCTTATATCC | TCGGCACAATCAAAATATC | FAM-CGGTTATTT/ZEN/  CACAAAAGACACGGTTGG-IABkFQ | wcw*A*_F_ | Downs *et al*. [3] |
| S. pneumoniae Serogroup 23A/B/F † | GGTGGACTTTCCGATGCAA | CACTGTCAACAAAAATGAGGTAATCTC | FAM-AAATGTCGGTATAGATAAAG-NFQ | wchV | Olwagen *et al.* [4] |
| S. pneumoniae Serotype 23B † | TTGAAGAAATTGCTCCAGAAACAT | CCAAAAGACTAGCCTCAACCACTAA | FAM-TAGAGCTATTTATCTTT  CGTGGTTTT-NFQ | wzx | Pholwat *et al.* [2] |
| S. pneumoniae Serotype 23F † | TGCTATTTGCGATCCTGTTCAT | AGAGCCTCCGTTGTTTCGTAAA | FAM-TTTCTCCGGCA  TCAAACGTTAAG-NFQ | wzy | Azzari *et al.* [1] |
| S. pneumoniae Serotype 24A † | CTTGGAGTTGCTAATTATGGGAAG | ATCTCTTACACGTGCACACTC | FAM-CACAGCATATCGTAA  AATACCCGCA-NFQ | wzx | Pholwat *et al.* [2] |
| S. pneumoniae Serogroup 24B/F ‡ | TCTGAAAGTAATTAG  TAAGATTAACGGAAG | TCCATCTACTTTTAAAATAGCTCCAAC | FAM-CCACAGTCCCAAAAT  TGTCAGCAACC-BHQ1 | wzy | Sakai *et al.* [5] |
| S. pneumoniae Serogroup 25A/F † | ATACCAACTAGAATCAGCAGGAC | AAATGGAATATCTTT  TGATAATTTACTCGC | VIC-CCGCTGGACTTACTGCAATA-NFQ | wcy*E* | Pholwat *et al.* [2] |
| S. pneumoniae Serogroup 25A/F/38 † | GTCTTACGTAGAACCTCTCTGGATGA | TGGTCCTACAAGCGACATGTG | FAM-TTGCCACAGATTTGG  AATATTTTGGTCGG-NFQ | wciI | Olwagen *et al.* [4] |
| S. pneumoniae Serotype 27 † | AGCGATTTAGCGACTGATATCC | TCTCAAAATCGATCTCGCGTG | FAM-TGTGGAAGGCGT  TTGAAGGTGACT-NFQ | wha*K* | Pholwat *et al.* [2] |
| S. pneumoniae Serogroup 28A/F † | CAACTACAGGTATTTTTGATATCGGAG | GTTTACTACGTTTGTGAAGCGC | FAM-AGAAAATAGTAGGTT  GATTGGCGGTGCT-NFQ | wcx*P* | Sakai *et al.* [5] |
| S. pneumoniae Serotype 29 † | TTCGAGTTGTGCCGTTTTTACA | GGCGTACCCACCTCTAAAATTTT | VIC-TGAATCCTAGTCTTTTCTCTGCG-NFQ | wcr*J* | Pholwat *et al.* [2] |
| S. pneumoniae Serotype 31 † | GCAGAAGTTTTAAGTCACGGAC | AGCATTACAGATGTCACTAAGGG | FAM-CCCCCACGTAAAACCGCAAGG-NFQ | wzy | Pholwat *et al.* [2] |
| S. pneumoniae Serogroup 32A/F † | GTACTTCCTGTTCTAGGCTTGG | CCCAGAGGAAAATAGCGTCTC | FAM-TTGTTCAAACC  CAACCACTGCTCC-NFQ | wzy | Sakai *et al.* [5] |
| S. pneumoniae Serogroup 33A/F/37 † | GGAACTGGTTCAGCAACTATACG | GGTTCTAAGACCGTCTGAAATACC | FAM-TAGGACTTTTCTGCCATGCC-NFQ | wzy | Pholwat *et al.* [2] |
| S. pneumoniae Serogroup 33B/C † | CCTGTTAGTGCACCTGTATTTAAC | GCATTCAAAACTCCTTCATCTCC | FAM-TTCGTTGTTCACGCCATTTA-NFQ | wci*N* | Pholwat *et al.* [2] |
| S. pneumoniae Serotype 33C † | CAGAGACAGTTTCAGCAAATCTTAG | AGCCTACACCTCTTATAAACGTTG | FAM-CCGTGTCCTATCCAC  AAACTTGTCTTCC-NFQ | Wzy | Sakai *et al.* [5] |
| S. pneumoniae Serotype 33D ‡ | CGTATAGTCTTGCGACATTTCA | TTCCACATGCGTTACCTCAC | FAM-CACAACTAG/ZEN/TTTTTTA  TCAAAAAGACCTTGGC-IABkFQ | wci*N* | Pholwat *et al.* [2] |
| S. pneumoniae Serogroup 34/37/17A † | GGATACTATGTACGAACAGATGGACTTG | CTCACTAACTCGCCCGAATAAAC | FAM-CCGACTATACTCCATTTGA-NFQ | wci*B* | Olwagen *et al.* [4] |
| S. pneumoniae Serotype 34 † | CGGTGGAGTAGGTCAAGATG | GTCTGTTCTCCCCAATATACTGAG | FAM-ACGGAGCGCCAATG  TACTTGAATAGTT-NFQ | Wzy | Pholwat *et al.* [2] |
| S. pneumoniae Serogroup 35A/C/42 † | TGTTTCAAGCTTCCCCTTTAGA | AAATGAAATCAAAGTATCACGTATCG | FAM-TTCAAAATACCCAG  GACACCCGTTCA-NFQ | wcr*K* | Pholwat *et al.* [2] |
| S. pneumoniae Serotype 35B † | GCATGGAGGTGGAGCATACA | TGTAAAGACTGCACAACTCGATATAAAA | FAM-CAATTTAAACAATATTAG  TAAAGCGCAGGTCAAGCAAA-NFQ | wcr*J* | Azzari *et al.* [1] |
| S. pneumoniae Serogroup 35F/47F † | GTGGTCGTATATACT  TGATGAATAAATCG | ACATACAAATTATCA  ACATACAGATAGGTC | FAM-TTCAACTGGTCGTCCGAATA-NFQ | Wzy | Pholwat *et al.* [2] |
| S. pneumoniae Serotype 36 † | CTTGTCTATTCAGCCCTTCTGG | CGCGATTATATTGTAAATTGGGAACT | VIC-AGAATGCCCGCTACAATGAG-NFQ | Wzy | Pholwat *et al.* [2] |
| S. pneumoniae Serotype 39 † | CAAAAAAATGAACTA  ACTCAAATAGTAACG | ATACTGTAATTTTCTTGTTTATTTGCGG | FAM-AAGTCAGGCGTATTC  TTCACAAGGGAAA-NFQ | wcr*G* | Pholwat *et al.* [2] |
| S. pneumoniae Serotype 41A † | GCAAATAGATGTATCCCAGTTAACAC | GGTAGCTCTTTTGGTTTAATGTCC | FAM-CGACCGAATAGTCT  AGCTTCAAAGG-NFQ | wci*B* | Pholwat *et al.* [2] |
| S. pneumoniae Serotype 41F † | TTTTTGGGAGGAAGTGCTTTT | AACCGCTTTCTCATGATTCATAACT | FAM-CTTCTGTGCTA  ACAGTGGAGAT-NFQ | Wzx | Pholwat *et al.* [2] |
| S. pneumoniae Serotype 43 † | AGAGGCTACATCAAATAGTTGGC | GAATCACACCGTAACTTCCAAAG | FAM-TCCAATAGTACTCA  CCCCTACCGAGC-NFQ | Wzx | Pholwat *et al.* [2] |
| S. pneumoniae Serotype 45 † | TCTAGCTACTTGACTA  AAATATTTGAACTG | GACGAGTCGATTTCGCTGTAT | VIC-CTTTTAGTGACCTCGCTCCC-NFQ | Wzy | Pholwat *et al.* [2] |
| S. pneumoniae Serotype 46 † | CGAAGTTTTTATATCTCTATTGGTTTG | TATCCCAGGAACTGGACGAA | FAM-TCATTCTTTCTTCAAT  TCCTTTCTGA-NFQ | Wzy | Sakai *et al.* [8] |
| S. pneumoniae Serogroup 47A/F † | AGGAATTGGTAGAGAGTTTGTGG | GAAAGTTGAACCATCATCCGTC | FAM-CACTTGATGGA  ATGCCTGCTGCC-NFQ | whaI | Pholwat *et al.* [2] |
| S. pneumoniae Serotype 48 † | CAGGTTTTGCTTCATATGGGAG | ATCGGCCAAAAGTTATCATTAGC | FAM-CGCTGCTTATGTGTA  TTACTCTCCCCTG-NFQ | Wzy | Sakai *et al.* [5] |
| Acintobacter baumanii † | TTTAGCTCGTCGTATTGGACT | CCTCTTGCTGAGGAGTAATTTT | FAM-TGGCAATGCAG  ATATCGGTACCCA-NFQ | blaOXA  -51-like | Gadsby *et al.*, [9] |
| Bordetella  holmesii † | GGCGACAGCGAGACAGAATC | GCCGCCTTGGCTCACTT | FAM-CGTGCAGATAGGCTT  TTAGCTTGAGCGC-NFQ | hIS*1001* | Tatti *et al.*, [10] |
| B. parapertussis † | TCGAACGCGTGGAATGG | GGCCGTTGGCTTCAAATAGA | FAM-AGACCCAGGGCGCACGCTGTC-NFQ | pIS*1001* | Tatti *et al.*, [10] |
| B. pertussis/  Holmesii † | CAAGGCCGAACGCTTCAT | GAGTTCTGGTAGGTGTGAGCGTAA | FAM-CAGTCGGCCTT  GCGTGAGTGGG-NFQ | IS*481* | Tatti *et al.*, [10] |
| B. pertussis/  bronchiseptica/ parapertussis † | CGCCAGCTCGTACTTC | GATACGGCCGGCATT | FAM-AATACGTCGAC  ACTTATGGCGA-NFQ | ptx*S1* | Tatti *et al.*, [10] |
| Escherichia coli † | GTCCAAAGCGGCGATTTG | CAGGCCAGAAGTTCTTTTTCCA | FAM-ACGGCAGAGAAGGTA-NFQ | uid*A* | Lee *et al.,*  [11] |
| Haemophilus influenzae † | CTCAGTTTCGTTTTATTACCA | CCAGTAACAACAAGGCTA | FAM-CGCATTCTTCTTCGTC  CATAACCTTC-NFQ | BexB | This Study |
| H. influenzae † | CTGAATTRGGYGATTATCTTTATGA | ACAATCAAAYTCAACHGAAAGHGA | FAM-AGGGATGAAA  GCYCGRCTTGCAT-NFQ | BexA | Maaroufi *et al,.*  [12] |
| H. influenzae † | CAAAATTGCCAAGATTAAATGCTT | TGCTCGCCATACTGCACAA | FAM-CCTGCGGTTAAACC-NFQ | IgA1 | Olwagen *et al.* [4] |
| H. influenzae,  type b † | TGTTCGCCATAACTTCATCTTAGC | CTTACGCTTCTATCTCGGTGATTAATAA | FAM-CACAAAACTTCTCAT  TCTTCGAGCCTA-NFQ | Bcs3` | Maaroufi *et al.,* [12] |
| Klebsiella pneumoniae ‡ | AGGCCGAATATGACGAAT | GGTGATCTGCTCATGAA | FAM-ACTACCGT  CACCCGCCACA-BHQ1 | glt*A* | Gadsby *et al.*, [9] |
| Morexella catarrhalis † | CCGCTTTTACAACCACTGCTT | TGTATCGCCTGCCAAGACAA | FAM-CAGCTGTTAGCCAGCC-NFQ | tonB | Olwagen *et al.* [4] |
| Neiserria  Lactamica † | TTGCCCGAGAACCATTGTATC | GCGGTTCTTATCACGTTCTATATTTG | FAM-TATTGGAGCGGACTAAA-NFQ | lacZ | Olwagen *et al.* [4] |
| Neiserria meningitidis † | GCACACTTAGGTGATTTACCTGCAT | CCACCCGTGTGGATCATAATAGA | FAM-CATGATGGCACAGCA  ACAAATCCTGTTT-NFQ | SodC | Dolan Thomas *et al.,*  [13] |
| Pneumocystis jiroveci ‡ | CCATCACATCTACGATTAC | GAACGAAATAACCATTGC | FAM-ACTCACATC  AACGAGGCGGT BHQ1 | Msg-A1 | Jensen, (Modified) [14] |
| Staphylooccus aureus † | GCTCAGCAAATGCATCACAAA | CACTATATACTGTTGGATCTTCAGAACCA | FAM-AGATAACGGCGTAAATA-NFQ | Tfp | Olwagen *et al.* [4] |
| Streptococcus pneumoniae ‡ | TCTTACGCAATCTAGCAGATGAAGC | GTTGTTTGGTTGGTTATTCGTGC | FAM-TTTGCCGAAAACG  CTTGATACAGGG- BHQ1 | LytA | McAvin *et al.*, [15] |
| S. pneumoniae ‡ | AGCAGGTGACTGGTAGGTAAC | CTCCTAATGCTGCTCC | FAM-CAGTTGCTT/ZEN/GCG  GTGCACTTG-IABkFQ | Xisco | This Study |
| S. pneumoniae † | AGCGATAGCTTTCTCCAAGTGG | CTTAGCCAACAAATCGTTTACCG | FAM-ACCCCAGCAAT  TCAAGTGTTCGCG-NFQ | Ply | Greiner *et al.* [16] |
| S. pneumoniae ‡ | CATTGGTGGCTTAGTAAGTGCAA | TACTAACACAAGTTC  CTGATAAGGCAAGT | FAM-TGTAAGCGG/ZEN/AAAAG  CAGGCCTTACCC-IABkFQ | PiaB | Trzciński *et al.* [17] |
| S. pyogenes † | GCACTCGCTACTATTTCTTACCTCAA | GTCACAATGTCTTGGAAACCAGTAAT | FAM-CCGCAACTCATCAAG  GATTTCTGTTACCA-NFQ | Spy | CDC 2008; Kodani, [18] |
| S. algalactidae † | GAACTCTAGTGGCTGGTGCATTG | GGAGTTGTCACTTGATCAGCATGT | FAM-ATTTTCACCAGCTGTATTAG-NFQ | Cfb | CDC [19] |
| S. oralis ‡ | ACCAGCAGATACGAAAGAAGCAT | AGGTTCGGGCAAGCGATCTTTCT | FAM-AAGGCTGCT/ZEN/GTT  GCTGAAGAAGT-IABkFQ | gtf*R* | Alvarez, [20] |
| Bacterial 16S ribosomal RNA gene ‡ | TCCTACGGGAGGCAGCAGT | GGACTACCAGGGTATCTAATCCTGTT | FAM-CGTATTACC/ZEN/GCG  GCTGCTGGCAC-IABkFQ | 16S | Nadkarni *et al.*, [21] |

Note: † Ordered from Thermofisher; ‡ ordered from Integrated DNA Technologies; NFQ is Nonfluorescent quencher; IABkFQ is an Iowa Black Fluorescent Quencher; BHQ1 is Black Hole Quencher 1.

Assessing the associations of nasopharyngeal carriage of selected microbes at enrolment

Logistic regression models of carriage (presence or absence) of SP, SA, MC, HI, *S. oralis* and HRV, are shown in Table S2. The participants with SP in their nasopharyngeal swabs are more likely co-carry MC and HI. Similarly, participants with MC carriage are more likely to co-carry SP and HI. HI carriage presence was positively associated with SP and MC. No significant associations were reported in the SA, S. oralis and HRV carriage with any microbes.

Table S2. Odds ratios with 95% confidence intervals for the interactions between different microbes in the nasopharynx.

| Variable | SP | SA | MC | HI | *S. oralis* | HRV |
| --- | --- | --- | --- | --- | --- | --- |
| SP |  |  |  |  |  |  |
| Absence | Reference | Reference | Reference | Reference | Reference | Reference |
| Presence (OR and CIs) | - | 1.9[0.6 -5.8] | **8.8 [4.5-17.0]** | **6.0 [3.4 -10.5]** | 3.4 [0.3 -33.8] | 2.7 [0.8 -8.6] |
| SA |  |  |  |  |  |  |
| Absence | Reference | Reference | Reference | Reference | Reference | Reference |
| Presence (OR and CIs) | 1.7 [0.6 -5.0] | - | 0.9 [0.3-3.0] | 0.5 [0.2 -1.6] | 2.7 [0.3 -28.1] | 1.6 [0.3 -8.0] |
| MC |  |  |  |  |  |  |
| Absence | Reference | Reference | Reference | Reference | Reference | Reference |
| Presence (OR and CIs) | **8.6 [4.5 - 16.6]** | 0.9[0.3 -3.0] | - | **2.4 [1.3 - 4.6]** | 1.2[0.3 -5.2] | 1.3 [0.5 -3.7] |
| HI |  |  |  |  |  |  |
| Absence | Reference | Reference | Reference | Reference | Reference | Reference |
| Presence (OR and CIs) | **6.0 [3.4 -10.4]** | 0.5[0.2 -1.6] | **2.4 [1.3 -4.5]** | - | Omitted | 1.3 [0.4 -3.8] |
| *S. oralis* |  |  |  |  |  |  |
| Absence | Reference | Reference | Reference | Reference | Reference | Reference |
| Presence (OR and CIs) | 3.6 [0.4 -33.3] | 2.2[0.2 -21] | 1.1 [0.3 -4.6] | Omitted | - | 2.5 [0.5 -13.9] |
| HRV |  |  |  |  |  |  |
| Absence | Reference | Reference | Reference | Reference | Reference | Reference |
| Presence (OR and CIs) | 2.5 [0.8 -8.1] | 1.7[0.3 -8.1] | 1.2 [0.4 -3.5] | 1.3 [0.4 -3.8] | 2.5[0.4 -14.7] | - |

**Note:** Significant associations (p < 0.05) are shown in bold

Table S3. Assessment of bacterial co-detection in HCLD+ participants

Assessment on whether bacterial co-detection in HCLD+ participants resulted from true interaction or chance is shown in Table S3. The analysis, based on expected values, indicates significant co-colonization of SP with HI (Observed [86/287] vs Expected [50.1/287], p <0.001); SP with MC (Observed [60/287] vs Expected [30.3/287], p <0.001) and MC with HI (Observed [54/287] vs Expected [32.4/287], p < 0.001]), affirming genuine interactions in the context of HCLD.

| **Bacterial co-detection** | **Observed Frequencies % (n/N)** | **Expected Frequencies %(n/N)** | ***P*- value** |
| --- | --- | --- | --- |
| SP with HI | 30% (86/287) | 17% (50.1/287) | **< 0.001** |
| SP with MC | 20.9% (60/287) | 10.6% (30.3/287) | **< 0.001** |
| SP with SA | 2.4% (7/287) | 2.4% (6.9/ 287) | 1.000 |
| MC with HI | 18.8% (54/287) | 11.3% (32.4/ 287) | **< 0.001** |
| MC with SA | 1% (3/287) | 1.5% (4.4/287) | 0.573 |
| SA and HI | 2.1% (6/287) | 2.5% (7.3/287) | 0.617 |

**Note:** Significant associations (p < 0.05) are shown in bold

### **SP serotype density detected at baseline in children with and without HCLD**

The median PCV-13 serotype density was similar between the HCLD+ and HCLD- groups, 8.5 log 10 GE/ml and 8.1 log10 GE/ml, respectively (Figure S1). Furthermore, the grouping of non-PCV13 serotypes showed no statistical difference in median densities between the HCLD+ and HCLD- groups.

Figure S1. Serotype density stratified into PCV 13 and non-PCV 13 serotypes from study participants at baseline

**Reference**

1. Azzari C, Moriondo M, Indolfi G, Cortimiglia M, Canessa C, Becciolini L, Lippi F, de Martino M, Resti M: Realtime PCR is more sensitive than multiplex PCR for diagnosis and serotyping in children with culture negative pneumococcal invasive disease. PLoS One 2010, 5(2):e9282,[<https://www.ncbi.nlm.nih.gov/pubmed/20174571>].

2. Pholwat S, Sakai F, Turner P, Vidal JE, Houpt ER: Development of a TaqMan Array Card for Pneumococcal Serotyping on Isolates and Nasopharyngeal Samples. J Clin Microbiol 2016, 54(7):1842-1850,[<https://www.ncbi.nlm.nih.gov/pubmed/27170020>].

3. Downs SL, Madhi SA, Van der Merwe L, Nunes MC, Olwagen CP: High-throughput nanofluidic real-time PCR to discriminate Pneumococcal Conjugate Vaccine (PCV)-associated serogroups 6, 18, and 22 to serotypes using modified oligonucleotides. Scientific Reports 2021, 11(1):23728,[<https://doi.org/10.1038/s41598-021-03127-9>].

4. Olwagen CP, Adrian PV, Madhi SA: Comparison of traditional culture and molecular qPCR for detection of simultaneous carriage of multiple pneumococcal serotypes in African children. Sci Rep 2017, 7(1):4628,[<https://doi.org/10.1038/s41598-017-04915-y>].

5. Sakai F, Sonaty G, Watson D, Klugman KP, Vidal JE: Development and characterization of a synthetic DNA, NUversa, to be used as a standard in quantitative polymerase chain reactions for molecular pneumococcal serotyping. FEMS Microbiol Lett 2017, 364(17),[<https://doi.org/10.1093/femsle/fnx173>].

6. Messaoudi M, Milenkov M, Albrich WC, van der Linden MPG, Bénet T, Chou M, Sylla M, Barreto Costa P, Richard N, Klugman KP, Endtz HP, Paranhos-Baccalà G, Telles J-N: The Relevance of a Novel Quantitative Assay to Detect up to 40 Major Streptococcus pneumoniae Serotypes Directly in Clinical Nasopharyngeal and Blood Specimens. PLoS One 2016, 11(3):e0151428,[<https://doi.org/10.1371/journal.pone.0151428>].

7. Downs SL, Madhi SA, van der Merwe L, Nunes MC, Olwagen CP: Optimization of a high-throughput nanofluidic real-time PCR to detect and quantify of 15 bacterial species and 92 Streptococcus pneumoniae serotypes. Scientific Reports 2023, 13(1):4588,[<https://doi.org/10.1038/s41598-023-31820-4>].

8. Sakai F, Chochua S, Satzke C, Dunne EM, Mulholland K, Klugman KP, Vidal JE: Single-plex quantitative assays for the detection and quantification of most pneumococcal serotypes. PLoS One 2015, 10(3):e0121064,[<https://www.ncbi.nlm.nih.gov/pubmed/25798884>].

9. Gadsby NJ, McHugh MP, Russell CD, Mark H, Conway Morris A, Laurenson IF, Hill AT, Templeton KE: Development of two real-time multiplex PCR assays for the detection and quantification of eight key bacterial pathogens in lower respiratory tract infections. Clinical Microbiology and Infection 2015, 21(8):788.e781-788.e713,[<https://www.sciencedirect.com/science/article/pii/S1198743X15004413>].

10. Tatti KM, Sparks KN, Boney KO, Tondella ML: Novel Multitarget Real-Time PCR Assay for Rapid Detection of &lt;span class=&quot;named-content genus-species&quot; id=&quot;named-content-1&quot;&gt;Bordetella&lt;/span&gt; Species in Clinical Specimens. Journal of Clinical Microbiology 2011, 49(12):4059,[<http://jcm.asm.org/content/49/12/4059.abstract>].

11. Lee D-Y, Shannon K, Beaudette LA: Detection of bacterial pathogens in municipal wastewater using an oligonucleotide microarray and real-time quantitative PCR. Journal of Microbiological Methods 2006, 65(3):453-467,[<https://www.sciencedirect.com/science/article/pii/S0167701205002812>].

12. Maaroufi Y, De Bruyne J-M, Heymans C, Crokaert F: Real-Time PCR for Determining Capsular Serotypes of Haemophilus influenzae. Journal of Clinical Microbiology 2007, 45(7):2305,[<http://jcm.asm.org/content/45/7/2305.abstract>].

13. Dolan Thomas J, Hatcher CP, Satterfield DA, Theodore MJ, Bach MC, Linscott KB, Zhao X, Wang X, Mair R, Schmink S, Arnold KE, Stephens DS, Harrison LH, Hollick RA, Andrade AL, Lamaro-Cardoso J, de Lemos APS, Gritzfeld J, Gordon S, Soysal A *et al*: sodC-Based Real-Time PCR for Detection of Neisseria meningitidis. PLoS One 2011, 6(5):e19361,[<https://doi.org/10.1371/journal.pone.0019361>].

14. Jensen L, Jensen AV, Praygod G, Kidola J, Faurholt-Jepsen D, Changalucha J, Range N, Friis H, Helweg-Larsen J, Jensen JS, Andersen AB: Infrequent detection of Pneumocystis jirovecii by PCR in oral wash specimens from TB patients with or without HIV and healthy contacts in Tanzania. BMC Infectious Diseases 2010, 10(1):140,[<https://doi.org/10.1186/1471-2334-10-140>].

15. McAvin JC, Reilly PA, Roudabush RM, Barnes WJ, Salmen A, Jackson GW, Beninga KK, Astorga A, McCleskey FK, Huff WB, Niemeyer D, Lohman KL: Sensitive and specific method for rapid identification of Streptococcus pneumoniae using real-time fluorescence PCR. J Clin Microbiol 2001, 39(10):3446-3451,[<https://www.ncbi.nlm.nih.gov/pubmed/11574554>].

16. Greiner O, Day PJR, Bosshard PP, Imeri F, Altwegg M, Nadal D: Quantitative Detection of Streptococcus pneumoniae in Nasopharyngeal Secretions by Real-Time PCR. Journal of Clinical Microbiology 2001, 39(9):3129-3134,[<https://jcm.asm.org/content/jcm/39/9/3129.full.pdf>].

17. Trzciński K, Bogaert D, Wyllie A, Chu MLJN, van der Ende A, Bruin JP, van den Dobbelsteen G, Veenhoven RH, Sanders EAM: Superiority of Trans-Oral over Trans-Nasal Sampling in Detecting Streptococcus pneumoniae Colonization in Adults. PLoS One 2013, 8(3):e60520,[<https://doi.org/10.1371/journal.pone.0060520>].

18. Kodani M, Yang G, Conklin LM, Travis TC, Whitney CG, Anderson LJ, Schrag SJ, Taylor TH, Beall BW, Breiman RF, Feikin DR, Njenga MK, Mayer LW, Oberste MS, Tondella MLC, Winchell JM, Lindstrom SL, Erdman DD, Fields BS: Application of TaqMan Low-Density Arrays for Simultaneous Detection of Multiple Respiratory Pathogens. Journal of Clinical Microbiology 2011, 49(6):2175,[<http://jcm.asm.org/content/49/6/2175.abstract>].

19. CDC: Centers for Disease Control and Prevention, Streptococcus Laboratory Protocols. Accessed 30-Jan-2016.

20. Àlvarez G, González M, Isabal S, Blanc V, León R: Method to quantify live and dead cells in multi-species oral biofilm by real-time PCR with propidium monoazide. AMB Express 2013, 3(1):1,[<https://doi.org/10.1186/2191-0855-3-1>].

21. Nadkarni MA, Martin FE, Jacques NA, Hunter N: Determination of bacterial load by real-time PCR using a broad-range (universal) probe and primers set. Microbiology 2002, 148(1):257-266,[<https://www.microbiologyresearch.org/content/journal/micro/10.1099/00221287-148-1-257>].
